# Structural but Not Functional Alterations in Cones in the Absence of the Retinal Disease Protein Retinitis Pigmentosa 2 (RP2) in a Cone-Only Retina

**DOI:** 10.3389/fgene.2019.00323

**Published:** 2019-04-05

**Authors:** Linjing Li, Kollu N. Rao, Hemant Khanna

**Affiliations:** Department of Ophthalmology and Visual Sciences, University of Massachusetts Medical School, Worcester, MA, United States

**Keywords:** retinal degeneration, cilia, ciliopathies, photoreceptors, opsin, cone

## Abstract

X-linked retinitis pigmentosa 2 (XLRP2) patients and *Rp2*^null^ mice exhibit severe cone photoreceptor degeneration. However, due to the paucity of cones in mammalian model systems, it is not clear how cones respond to the loss of RP2. Here we have used the *Nrl^-/-^* mice, which develop a rodless and short wavelength (S) opsin-containing cone-only retina, to generate *Rp2*^null^::*Nrl^-/-^* double knock out (*Rp2*-DKO) mice. We found that the ciliary axoneme and the outer segments (OSs) of the cones were significantly longer with disorganized membrane infoldings as compared to the *Nrl^-/-^* mice. Additionally, we found misregulation in the expression of the genes related to ophthalmic disease, cell trafficking, and stress-response in the *Rp2*-DKO mice prior to the onset of cone degeneration. Surprisingly, the loss of RP2 did not affect progressive photoreceptor dysfunction of the *Nrl^-/-^* mice and the trafficking of S opsin. Our data suggest that RP2 is a negative regulator of cone OS length but does not affect S-opsin trafficking and S-cone function. Our studies also provide a cone-only platform to design cone-targeted therapeutic strategies for X-linked RP2.

## Introduction

Cilia are evolutionarily conserved microtubule-based antennae involved in regulating a myriad of cellular signaling cascades (Singla and Reiter, 2006; Gerdes et al., 2009). During cilia formation, the basal body (mother centriole) docks at the apical plasma membrane and initiates the nucleation of ciliary microtubules in the form of transition zone and distal axoneme (Inglis et al., 2006). The light-sensing outer segment (OS) of the rod and cone photoreceptors is a modified sensory cilium. Although the machinery involved in the generation of the photosensory cilia is conserved, the photoreceptors develop an elaborate distal cilium in the form of multiple membranous disks loaded with billions of opsin molecules and lipids involved in the phototransduction cascade (Rosenbaum et al., 1999; Rosenbaum and Witman, 2002; Besharse et al., 2003; Pazour and Witman, 2003; Insinna et al., 2009; Kennedy and Malicki, 2009). Even subtle defects in the formation or function of the photoreceptor cilia are associated with retinal degenerative diseases (Besharse et al., 2003; Anand and Khanna, 2012; Deretic and Wang, 2012; Yildiz and Khanna, 2012).

The cone and rod OSs are morphologically and physiologically different from each other (Kandel, 2013), indicating that they have developed unique pathways to generate and maintain the sensory cilia. Functionally, the rods are sensitive to very low light intensities whereas cones mediate majority of our vision due to their ability to function in high light intensities. Hence, in retinal degenerative diseases, it is the loss of cones that affects the quality of life of patients (Kandel, 2013). Whereas the rod OSs are loaded with rhodopsin, two types of cone opsins are expressed in mice: red/green (medium wave-length, M)-opsin or short wave-length S-opsin. Moreover, the rod OSs have discrete disks independent of the OS (ciliary) plasma membrane loaded with rhodopsin, the cone OS invaginates and is continuous with the plasma membrane (Deretic and Wang, 2012).

We previously showed that the deletion of the retinal ciliopathy protein retinitis pigmentosa 2 (RP2) (Schwahn et al., 1998) results in hyperelongation of the ciliary microtubules and defective OS structure and function of M-opsin expressing cones but not rod photoreceptors in mice (Li et al., 2013, 2015). However, due to the paucity of cones in the mammalian retina, it has been difficult to understand how the loss of RP2 specifically modulates cone OS extension in mice and leads to cone dysfunction. To tackle this roadblock, we have used the *Nrl^-/-^* mice, which develop a rodless and cone-enriched retina. The cones of the *Nrl^-/-^* mice express cone-specific genes and exhibit morphological and physiological features of wild type (WT) cones (Mears et al., 2001; Daniele et al., 2005). These mice have been previously used to assess cone-related pathogenesis in retinal degenerative diseases (Chakraborty et al., 2010; Cideciyan et al., 2011; Thapa et al., 2012; Rajala et al., 2013; Rao et al., 2016). The *Nrl^-/-^* mouse platform can also be used to understand the responses of foveal cones that are present in a rodless environment in the primates (Roger et al., 2012). The aim of the current study was to understand how cones respond to the loss of RP2 in a rodless environment.

## Materials and Methods

### Mice

All animal experiments were performed in accordance with the approved procedures of the Institutional Animal Care and Use Committee. All mice were maintained in the same conditions of 12-h light to 12-h dark, with unrestricted access to food and water. Lighting conditions were kept constant in all cages with illumination of 10 to 15 lux at the level of the cages.

The *Rp2*-DKO mice were generated by breeding the homozygous *Nrl^-/-^* with the previously reported *Rp2*^null^ mice (Mears et al., 2001; Li et al., 2013). Male mice of appropriate genotypes were used in these studies. All mice were genotyped to confirm the absence of the *rd8* allele.

### Antibodies

Antibodies against β-tubulin, actin and acetylated α-tubulin were obtained from Sigma-Aldrich (St. Louis, MO, United States). Peanut agglutinin (PNA) was procured from Vector Labs (Burlingame, CA, United States); Rhodopsin and S-opsin antibodies were purchased from Millipore (Billerica, MA, United States) and SantaCruz, respectively.

### Fundus Examination and Electroretinography (ERG)

The fundus and ERG recordings were performed essentially as described earlier (Chang et al., 2006; Rao et al., 2016). Only photopic (cone-mediated) ERGs were recorded as the *Nrl^-/-^* mice develop a cone-only retina.

### Immunofluorescence and Transmission Electron Microscopy (TEM)

For immunofluorescence analyses, mouse eyes were enucleated and then fixed in 4% paraformaldehyde in PBS (pH 7.4) followed by cryosectioning and staining as recently described (Li et al., 2013). Primary antibodies were prepared in blocking solution and slides were further incubated overnight at 4°C. Sections were then washed three times with PBS and incubated for 1 h with goat anti-rabbit (or mouse) Alexa Fluor 488, 546, or 647 nm secondary antibody (1:500) at RT. Hoechst 33342 (Life Technologies, Corp.) was used to label the nuclei. The sections were then visualized using a scanning confocal microscope (Leica TCS SP5 II laser; Leica Microsystems).

For transmission electron microscopy (TEM), mouse eyes were enucleated and fixed in 2% glutaraldehyde, 2% paraformaldehyde in 0.1 M sodium cacodylate buffer (pH 7.2) overnight at RT. The anterior portion was removed on the next morning and processed as described (Li et al., 2013). Ultrathin sections were observed in a Zeiss EM900 electron microscope and pictures were taken with a coupled digital camera using the ImageSP software.

### Transcriptomic Analysis

Total RNA from the *Nrl^-/-^* and *Rp2*-DKO mice was extracted using the RNeasy plus mini kit (Qiagen, United States) according to manufacturer’s instructions. The sample from neuroretina plus RPE was isolated from six retinas of each genotype in triplicates. The transcriptomic profile and data analysis of the differentially expressed transcripts were analyzed at Beijing Genomics Institute (Hong Kong), as described. Subsequent bioinformatic analysis was performed using the RPKM (reads per kilo base mapped) values, and the data were analyzed as log_2_ ratio from the RPKM values. We selected genes that were significantly altered at least 1.5-fold. Pathway analysis was performed using the IPA system (Ingenuity Systems, Redwood City, CA, United States). The differentially expressed genes were validated by qRT-PCR, as described (Rao et al., 2016).

## Results

### Characterization of the *Rp2*-DKO Mice

**FIGURE 1:**
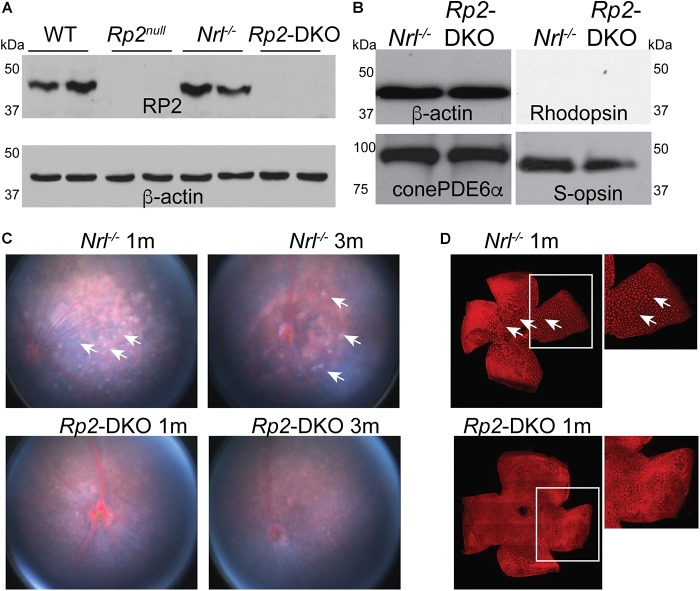
Characterization of the *Rp2*-DKO mice. **(A,B)** Retinal extracts (100 μg) from the mice of the indicated genotypes were analyzed by SDS-PAGE and immunoblotting using anti-RP2, rhodopsin, S-opsin, cone PDE6α, and β-actin (loading control) antibodies. Fundus **(C)** and flat mounted retinal staining **(D)** of the *Nrl^-/-^* and *Rp2*-DKO mice was performed. Arrows point to the white spots in the fundus photograph that correspond to the whorls and rosettes observed in the PNA (red; peanut agglutinin) stained flat mounted retinas of the *Nrl^-/-^* mice. Such spots are undetectable in the *Rp2*-DKO mouse retinas.

We previously showed that the ablation of *Rp2* in mice (*Rp2*^null^) results in cone OS extension and progressive cone dysfunction (Li et al., 2015). However, due to the overwhelming majority of rods in the mouse retina, it is difficult to investigate how cones respond to the loss of RP2. We therefore, generated *Rp2*^null^:*Nrl^-/-^* double knockout (*Rp2*-DKO) mice and confirmed the absence of RP2 by immunoblotting using an anti-RP2 antibody. We detected the RP2-immunoreactive band in the WT and the *Nrl^-/-^* mice but not in the *Rp2*^null^ or the *Rp2*-DKO mice (Figure 1A). We also tested whether the loss of RP2 in cones resulted in alterations in the expression of other cone-specific phototransduction genes. We did not detect any differences in the expression of cone phosphodiesterase and S- opsin between the *Rp2*-DKO and *Nrl^-/-^* mouse retinas. As predicted, rhodopsin expression was undetectable in these retinas (Figure 1B).

We next assessed the retinal morphology of the *Rp2*-DKO mice. Whereas the fundus examination of the *Nrl^-/-^* mice at 1 and 3 months of age revealed the presence of white spots, which represent whorls and rosettes present in the outer nuclear layer (Roger et al., 2012), the *Rp2*-DKO mice did not exhibit such structures (Figure 1C). These findings were corroborated in the flat mounted retinas stained with peanut agglutinin (PNA; marker of cone extracellular matrix; red) (Figure 1D).

### Cone Function and Morphology in the *Rp2*-DKO Mice

**FIGURE 2:**
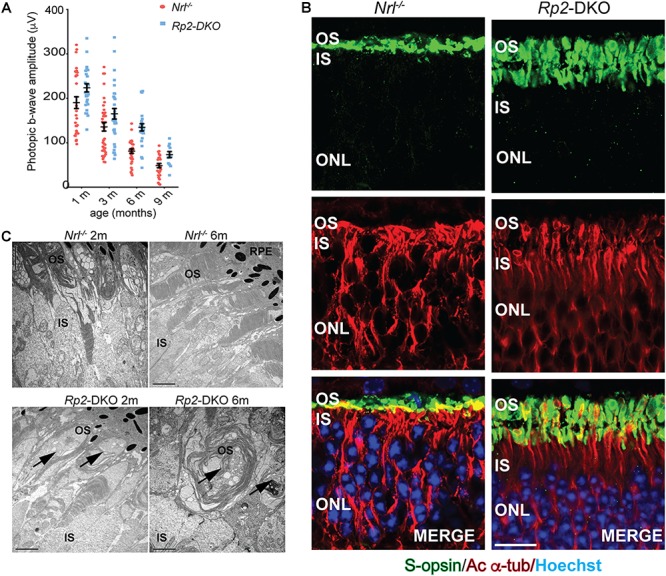
Phenotypic analysis of the *Rp2*-DKO mice: **(A)** Photopic b-wave analysis of the *Nrl^-/-^* and *Rp2*-DKO mice at the indicated ages did not reveal statistically significant differences. The data represent Standard Error of Mean. **(B)** Immunofluorescence analysis of the cryosections of 3 months old *Nrl^-/-^* and *Rp2*-DKO mouse retinas was performed using anti-S-opsin (green) and acetylated α-tubulin (red) antibodies. Nuclei were stained with Hoechst (blue). OS, outer segment; IS, inner segment; ONL, outer nuclear layer. Scale bar: 25 μm. **(C)** TEM analysis of *Nrl^-/-^* and *Rp2*-DKO mouse retinas at 2 and 6 months of age was performed. Arrows indicate the irregular outer segment morphology in the *Rp2*-DKO mice. RPE, retinal pigment epithelium. Scale bar: 2 μm.

It was previously reported that the *Nrl^-/-^* mice exhibit cone-mediated supranormal photopic b-wave responses up to ∼3 months of age (Roger et al., 2012). We therefore, analyzed the effect of loss of RP2 on cone function in these mice. We found that the loss of RP2 did not affect the supranormal responses of the *Nrl^-/-^* mice. Moreover, the rate of age-dependent decline in photopic responses of the *Nrl^-/-^* mice was indistinguishable from the *Rp2*-DKO mice (Figure 2A). These data indicated that the previously reported cone dysfunction observed in the *Rp2*^null^ mice or in the mice in which the *Rp2* gene was conditionally ablated specifically in M-opsin expressing cones was largely due to their dysfunction (Li et al., 2013, 2015). The *Rp2*-DKO mice have an overwhelming majority of short wave-length S-opsin expressing cones, which could mask the effect on the M-opsin expressing cones.

As the *Rp2*-DKO mice did not exhibit major changes in photoreceptor dysfunction, we next assessed whether the long cone OS phenotype of the *Rp2*^null^ mice is phenocopied by the *Rp2*-DKO mice. Immunostaining of the *Rp2*-DKO mouse retinas with acetylated α-tubulin (microtubules) and S-opsin antibodies (outer segment marker) revealed the presence of elongated cone OS at 3 months of age as compared to the *Nrl^-/-^* mice (Figure 2B). Furthermore, we did not detect significant differences in the localization of S-opsin between the *Nrl^-/-^* and the *Rp2*-DKO retinas. The elongated OSs were detectable even at earlier stages (data not shown).

These results suggested that the longer cone OSs are functional in the S-cone rich *Rp2*-DKO mice. Thus, we predicted that the morphology of these cones would also be comparable to the *Nrl^-/-^* mice. However, ultrastructural analysis demonstrated the presence of abnormal OS membrane vesicles in the OSs in 2 months old *Rp2*-DKO mice (Figure 2C). As previously described, the *Nrl^-/-^* mice showed membranous vesicles in the OSs over wide areas of the retina and shorter OSs but with still discernible OS infoldings at 6 months of age (Roger et al., 2012). In contrast, the 6 months old *Rp2*-DKO mice exhibited abnormal infoldings loaded with membranous vesicles (Figure 2C). The connecting cilium appeared normal in the micrographs (data not shown).

### Retinal Gene Expression Profile of *Rp2*-DKO Mice

**FIGURE 3:**
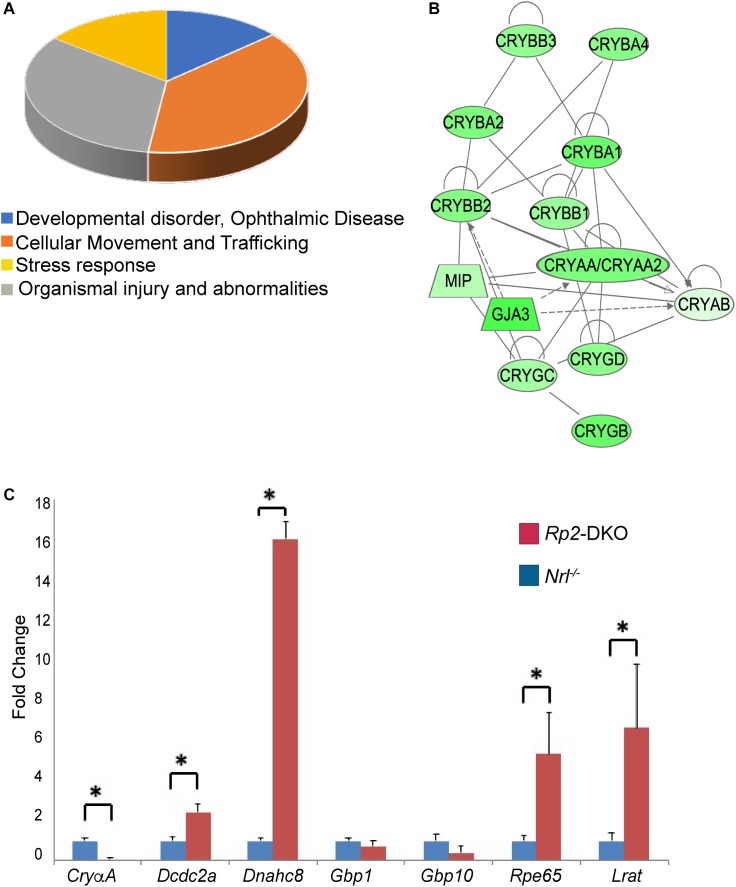
Pathways dysregulated in the *Rp2*-DKO mice. **(A)** Pie chart representation of the major pathways that are represented by the genes with altered expression in the *Rp2*-DKO mice as compared to the *Nrl^-/-^* mice. **(B)** Network construction using IPA revealed the crystallin subunits as the major representative altered network in the *Rp2*-DKO mice. **(C)** qRT-PCR analysis of the indicated genes was performed using three independent samples each with six retinas. ^∗^*p* < 0.0001.

The absence of rods in the *Nrl^-/-^* mice provides a unique opportunity to investigate pure cone responses to the loss of a retinal disease gene. To delineate the cellular responses underlying the intriguing phenotypes of elongated cone OSs, we performed comparative transcriptomic analysis of the retinas from the *Rp2*-DKO and *Nrl^-/-^* mice at 1 month of age. We chose this age to avoid degeneration-associated artifacts. We compared six retinas from each genotype and repeated the experiment three times. Our analysis revealed 154 differentially expressed genes (>1.5 fold) in the *Rp2*-DKO mice as compared to the *Nrl^-/-^* mice (Supplementary Table S1). These genes represented major biological pathways, including developmental and ophthalmic disorders, cellular movement/trafficking, and stress responses (Figure 3A and Supplementary Table S2). We identified several crystallin subunits whose expression was downregulated in the *Rp2*-DKO mice (Figure 3B). The crystallin subunits belong to the chaperone family of proteins, respond to stress and inflammation and are known to be upregulated during retinal disease (Fort and Lampi, 2011). On the other hand, *Lrat* (lecithin retinol acyltransferase) *Rpe65* (retinal pigment epithelium 65), *Dcdc2a* (Doublecortin domain containing protein), and *Dnahc8* (dynein axonemal heavy chain 8) were the major representative genes of the ophthalmic disease and cilia length regulation and cell trafficking pathways (Redmond et al., 1998; Ruiz et al., 1999; Moiseyev et al., 2005; Samant et al., 2005; Grati et al., 2015). We therefore validated the expression of these genes by qRT-PCR. As shown in Figure 3C, whereas the expression of *Cry*α*A* subunit was diminished in the *Rp2*-DKO mice, *Dnahc8* exhibited ∼16-fold increase in expression. *Lrat* and *Rpe65* expression was also elevated by ∼5 and 7 fold, respectively. As control, Gbp1 (guanylate binding protein 1) and Gbp10 did not show any change in the expression levels in the RNAseq and qRT-PCR analyses.

## Discussion

The mechanisms by which cones respond to the loss of a retinal disease genes have remained elusive. We had previously reported that the loss of RP2 results in OS elongation specifically of cones. Here we report that the elongation of the cone OSs is associated with an upregulation of the genes involved in the phototransduction cascade and microtubule-associated and intracellular trafficking and cell migration pathways. Significant decrease in the stress response related genes with reduction of the whorls and rosette formation in the *Nrl^-/-^* mice was also observed in the absence of RP2. However, no effect on the progression of the age-dependent decline in photoreceptor function of the *Nrl^-/-^* mice or the localization of S-opsin was observed in the absence of RP2. Given that the majority of cones in the *Nrl^-/-^* mice are S-cones, our data suggest that elongation of the S-cone OSs is independent of the localization of S opsin, indicating a distinct regulation of the structural and functional pathways of the cones.

The whorls and rosettes are believed to be formed due to the structural collapse of the retina in the absence of rods. This leads to a detachment of the cones from the retinal pigment epithelium (RPE) (Roger et al., 2012). Our data suggest that the elongation of the cone OSs led to the dissolution of the whorls and rosettes due to their renewed contact with the RPE. However, the lack of the whorls and rosettes and a reduction in the stress response pathways in the *Rp2*-DKO mice did not perturb the age-dependent decline in photoreceptor function, further indicating a distinction between the structure of the cone OS and ERG outcomes. Additional studies are needed to investigate this intriguing observation.

Not much is known about the mechanism of cone OS extension. It is possible that RP2 is involved in modulating the turnover of tubulin required for the extension of the axoneme. Support of this hypothesis comes from the analysis of the primary structure of RP2. RP2 shows homology to tubulin-binding cofactor C and was found to stimulate the activity of tubulin as well as another small GTPase ARL3 (Arf-GTPase like-3) (Bartolini et al., 2002; Grayson et al., 2002; Kuhnel et al., 2006). Moreover, the upregulation of *Dnahc8, Dnai1*, and *Dcdc2a* (Figure 3C and Supplementary Table S1), microtubule-associated protein encoding genes suggests a possible role of RP2 in structural regulation of the cilia. Although DNAHC8 is known to be involved in modulating motile cilia function (Samant et al., 2005), it may play a different and as yet unknown role in the retina. An alternative explanation for long cone OS is that RP2 regulates the turnover of the OS components in cones. Our observations that the elongated OSs show deformed membrane extensions suggest that RP2 is potentially involved in the availability of the proteins involved in facilitating cone OS shedding by regulating their trafficking to the OS.

It was previously reported that the *Nrl^-/-^* mice exhibit Müller cell activation, retinal vasculature defects, and RPE atrophy (Roger et al., 2012). These pathways may still be contributing to the observed decline in photoreceptor function in the *Rp2*-DKO mice. Additional investigations are necessary to determine the involvement of these pathways in cone dysfunction in RP2-associated as well as other retinopathies.

## Ethics Statement

All animal experiments were performed in accordance with the approved procedures of the Institutional Animal Care and Use Committee.

## Author Contributions

LL and KR performed the research. HK designed the research and wrote the manuscript.

## Conflict of Interest Statement

The authors declare that the research was conducted in the absence of any commercial or financial relationships that could be construed as a potential conflict of interest.

## Supplementary Material

TABLE S1 Differentially expressed genes in the *Rp2*-DKO mice.

TABLE S2 Representative pathways that are altered in the *Rp2*-DKO mice.
